# HHL1 and SOQ1 synergistically regulate nonphotochemical quenching in Arabidopsis

**DOI:** 10.1016/j.jbc.2023.104670

**Published:** 2023-04-05

**Authors:** Sujuan Duan, Beibei Dong, Ziqi Chen, Liu Hong, Pengxiang Zhang, Ziyue Yang, Hong-Bin Wang, Hong-Lei Jin

**Affiliations:** 1 Institute of Medical Plant Physiology and Ecology, School of Pharmaceutical Sciences, Guangzhou University of Chinese Medicine, Guangzhou, China; 2 State Key Laboratory of Biocontrol and Guangdong Provincial Key Laboratory of Plant Resources, School of Life Sciences, Sun Yat-sen University, Guangzhou, China; 3 Key Laboratory of Chinese Medicinal Resource from Lingnan (Guangzhou University of Chinese Medicine), Ministry of Education, Guangzhou, China; 4 State Key Laboratory of Dampness Syndrome of Chinese Medicine, Guangzhou University of Chinese Medicine, Guangzhou, China; 5 Guangzhou Key Laboratory of Chinese Medicine Research on Prevention and Treatment of Osteoporosis, The Third Affiliated Hospital of Guangzhou University of Chinese Medicine, Guangzhou, China

**Keywords:** nonphotochemical quenching, photosynthesis, photoprotection, photo-damage repair, protein–protein interaction

## Abstract

Nonphotochemical quenching (NPQ) is an important photoprotective mechanism that quickly dissipates excess light energy as heat. NPQ can be induced in a few seconds to several hours; most studies of this process have focused on the rapid induction of NPQ. Recently, a new, slowly induced form of NPQ, called qH, was found during the discovery of the quenching inhibitor suppressor of quenching 1 (SOQ1). However, the specific mechanism of qH remains unclear. Here, we found that hypersensitive to high light 1 (HHL1)—a damage repair factor of photosystem II—interacts with SOQ1. The enhanced NPQ phenotype of the *hhl1* mutant is similar to that of the *soq1* mutant, which is not related to energy-dependent quenching or other known NPQ components. Furthermore, the *hhl1 soq1* double mutant showed higher NPQ than the single mutants, but its pigment content and composition were similar to those of the wildtype. Overexpressing *HHL1* decreased NPQ in *hhl1* to below wildtype levels, whereas NPQ in *hhl1* plants overexpressing *SOQ1* was lower than that in *hhl1* but higher than that in the wildtype. Moreover, we found that HHL1 promotes the SOQ1-mediated inhibition of plastidial lipoprotein through its von Willebrand factor type A domain. We propose that HHL1 and SOQ1 synergistically regulate NPQ.

Plants absorb light energy and convert it into chemical energy through photosynthesis, which provides energy for the growth and reproduction of plants and ultimately most living organisms (1). Light energy is required for plant growth, but excessive light energy increases the production of intracellular reactive oxygen species, resulting in oxidative damage and impeding normal plant growth (2, 3, 4). Because plants are immobile and cannot avoid adverse light conditions, they have evolved systematic light protection mechanisms to cope with frequent fluctuations in light levels (5). Photoprotection in plants primarily involves two strategies: (1) reducing the absorption of light energy and (2) dissipating excess light energy (6).

Nonphotochemical quenching (NPQ), a process that dissipates excess light energy as heat, is an important photoprotective mechanism due to its fast and efficient nature (7). NPQ can be divided into different components based on the molecules utilized and the time required. Energy-dependent quenching (qE), the best-studied component of NPQ, is rapidly induced and released (within seconds to min) (8, 9). Key players in qE include thylakoid membrane proton gradients (△pH), PsbS, and the xanthophyll cycle (10). Zeaxanthin-dependent quenching (qZ) is produced and discharged in a few to 10 min; this process depends on zeaxanthin but is independent of PsbS and the proton gradient once zeaxanthin is present (11). State transitions (qT) reduce excessive excitation pressure on photosystem II (PSII) in plants and algae by moving light harvesting complex II (LHCII) antennas from PSII to PSI, where excitation energy is suppressed by PSI, resulting in qT (12). Photoinhibitory quenching (qI) takes several hours or more to produce and is therefore also referred to as sustained quenching. The inactivation and degradation of the PSII core protein D1 is one (but not the only) cause of qI (13).

A recently proposed additional quenching mechanism involves a continuous decrease in the fluorescence yield of the major antenna complex LHCII. This photoprotective, slowly reversible process is induced by plastidial lipoprotein (LCNP) and is negatively regulated by the quenching inhibitor suppressor of quenching 1 (SOQ1) (13, 14). SOQ1 was identified in a screen for suppressors of *nonphotochemical quenching 4* in the Arabidopsis (*Arabidopsis thaliana*) *npq4* mutant lacking PsbS. The formation of NPQ in the *soq1* mutant is dependent on light intensity and exhibits slow relaxation kinetics (14). Because the characteristics of NPQ in *soq1* distinguish it from known NPQ components, such as PsbS, zeaxanthin, pH gradient formation, or the chloroplast thylakoid protein kinase STN7 (9, 14), researchers named this NPQ component qH (for its position in the alphabet; photoprotective quenching (“H”) comes before quenching due to PSII photodamage (“I”), which is another slowly induced form of NPQ). qH occurs in the antenna, specifically in the peripheral antenna of PSII (15).

SOQ1 is a chloroplast-localized thylakoid membrane protein that contains a thioredoxin-like domain on the lumenal side of the thylakoid membrane (14). SOQ1 prevents qH through inhibition of LCNP under nonstress conditions (13). Because SOQ1 contains a thioredoxin-like domain in the lumen, it might maintain its target(s) (such as LCNP) in a reduced state. However, the altered mobility of higher molecular mass LCNP proteins in *soq1* was not reversed by the addition of dithiothreitol. Therefore, whether this domain is redox-active and functions by reducing or oxidizing its target(s) remains to be determined (13). Recently, the structural, genetic, and biochemical characterization of Arabidopsis SOQ1 lumenal domains was resolved and revealed that the C-terminal region of SOQ1 is essential for negatively regulating qH through interacting with and providing reducing power to its target proteins (possibly LCNP or a protein upstream of LCNP) (16). The chloroplast protein hypersensitive to high light 1 (HHL1) acts as a photodamage repair factor of PSI. HHL1 deficiency results in hypersensitivity to high light, and *hhl1* mutants exhibit high NPQ activity through accelerating the degradation of PSII core subunits under high light, decreasing the accumulation of PSII core subunits and PSII–LHCII supercomplexes. HHL1 localizes in the stroma-exposed thylakoid membrane and associates with the PSII core monomer complex through direct interaction with PSII core proteins CP43 and CP47 (17). As mentioned above, qI is attributed to processes involving photodamage of PSII. Some reports suggest that HHL1 is involved in regulating qI, but there is no direct evidence for this (15, 18).

In the current study, to elucidate the mechanism by which HHL1 regulates NPQ, we performed yeast two-hybrid screening of an Arabidopsis cDNA library using HHL1 as bait. We identified SOQ1 as an interacting protein of HHL1, suggesting that HHL1 may coordinate with SOQ1 to regulate NPQ. Our identification of the synergistic functions of HHL1 and SOQ1 sheds light on the regulatory mechanism of plant NPQ and provides a theoretical basis for how to maximize the balance of light absorption, photoprotection, and photosynthesis efficiency.

## Results

### NPQ is enhanced in the hhl1 mutant under growth-light conditions but is independent of PsbS

**Figure 1:**
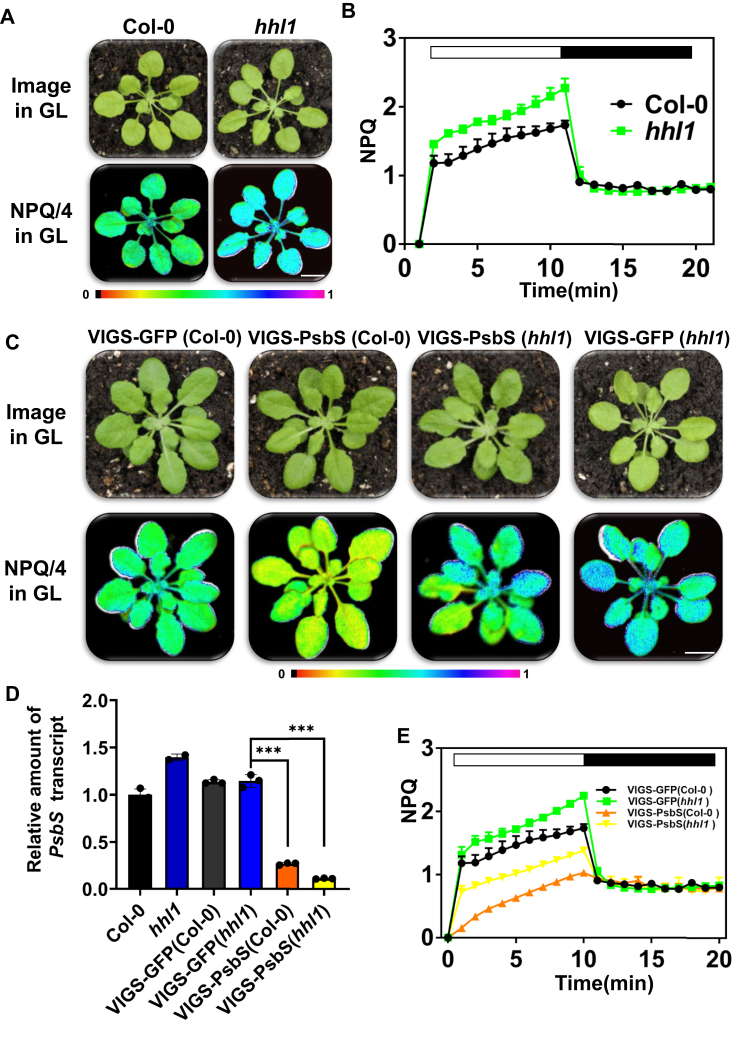
**NPQ induction and relaxation in Col-0, *hhl1*, VIGS-PSBS (Col-0), and VIGS-GFP (*hhl1*), NPQ induction curves of 4-week-old Arabidopsis Col-0, *hhl1*, VIGS-GFP(Col-0), VIGS-PSBS (Col-0), VIGS-GFP (*hhl1*) and VIGS-PSBS (*hhl1*) grown under growth-light conditions, induced by treatment with actinic light at 500 μmol photons·m**^**−2**^**·s**^**−1**^**for 10 min, and relaxed in the dark for 10 min.***A*, the saturation pulse was applied every 30 s. *C*, false-colored image of NPQ in plants after 10 min of actinic light treatment. Scale bar, 1 cm. *B* and *E*, NPQ kinetics curve. *D*, silencing efficiency of *PsbS* in VIGS plants, means ± SD, Error bars represent SD of three biological repeats (∗∗∗*P* < 0.001, Student’s t test). NPQ, nonphotochemical quenching; VIGS, virus-induced gene silencing.

Under growth-light conditions, the *hhl1* mutant possessed much higher NPQ activity than wildtype (WT) plants (Fig. 1, *A* and *B*) (17), but the role of HHL1 in NPQ is unclear. The light-response curves of PSII quantum yield (φPSII) and the electron transfer rate did not change between WT and *hhl1* under growth-light conditions (17). The high NPQ in *hhl1* is probably related to photoprotection. NPQ is triggered by pH, either directly by the protonation of antenna components (*e.g.*, PsbS) or indirectly by xanthophyll cycle enzymes (7). Therefore, to examine the relationship between the enhanced NPQ observed in *hhl1* and known components of NPQ, we generated Col-0 and *hhl1* Arabidopsis lines silencing PsbS *via* Tobacco rattle virus–based virus-induced gene silencing (VIGS) to determine whether qE is affected in plants with HHL1 deficiency. Reverse transcription quantitative PCR analysis confirmed the inactivation of *PsbS* expression (by >80%) in VIGS-PsbS (*hhl1*) plants relative to the WT (Fig. 1*D*). VIGS-PsbS (Col-0) and VIGS-PsbS (*hhl1*) exhibited lower levels of NPQ than the WT. However, the enhanced NPQ in *hhl1* was not abolished in VIGS-PsbS (*hhl1*) plants (Fig. 1, *C* and *D*), suggesting that quenching attributed to *hhl1* does not depend on PsbS.

Because NPQ occurred in the *hhl1* mutant in the absence of PsbS, we tested whether HHL1-dependent NPQ requires the formation of a pH gradient across the thylakoid membrane. The uncoupler nigericin prevents the acidification of the thylakoid lumen and consequently inhibits qE. When we infiltrated *hhl1* leaves with nigericin, NPQ remained high (Fig. S1), suggesting that quenching attributed to *hhl1* is not related to qE or the pH gradient.

### HHL1 physically interacts with SOQ1

To explore how HHL1 performs its function, we designed a yeast two-hybrid screen of a normalized Arabidopsis cDNA library using an HHL1 protein with a truncated chloroplast transit peptide (cTP) as the bait. We identified eight chloroplast proteins as possible interactors of HHL1 (Table S1). Among them was SOQ1, encoded by the At1g56500 locus, that negatively regulates a light intensity–dependent, slowly reversible form of NPQ (13, 14, 19).

**Figure 2:**
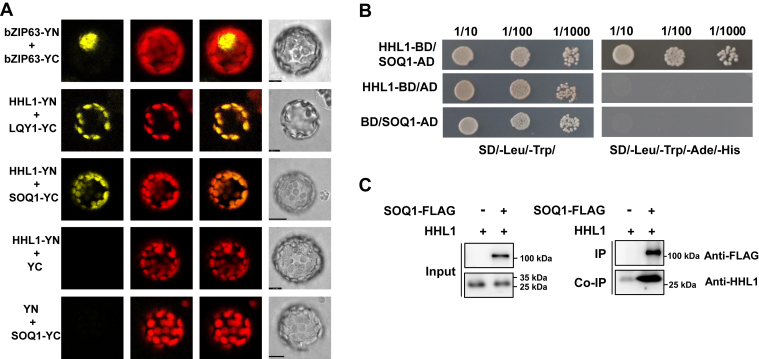
**BiFC and yeast two-hybrid (Y2H) analysis of the interaction of HHL1 and SOQ1.***A*, BiFC assay. *HHL1* was cloned into the YN vector, and *SOQ1* was cloned into the YC vector. The vectors were cotransformed into Arabidopsis protoplasts, and fluorescence was observed by confocal microscopy. bZIP63-YN + bZIP63-YC and HHL1-YN + LQY1-YC were used as positive controls; YN and SOQ1-YC were used as the negative control. Scale bar, 10 μm. *B*, Y2H analysis. *HHL1* was cloned into the BD vector to form a fusion protein expression vector (HHL1-BD), and *SOQ1* was cloned into the AD vector to form a fusion protein expression vector (SOQ1-AD). HHL1-BD and SOQ1-AD were cotransformed into yeast strain Y2H Gold; HHL1-BD and AD, and SOQ1-AD and BD were used as negative controls. *C*, the 10-day-old transgenic Arabidopsis seedlings expressing SOQ1-FLAG and the purified His-HHL1 fusion protein were used for the Co-IP assay. Two additional independent biological replicates were performed with similar results. BiFC, bimolecular fluorescence complementation; Co-IP, co-immunoprecipitation; HHL1, hypersensitive to high light 1; SOQ1, suppressor of quenching 1.

To confirm if there is a functional relationship between HHL1 and SOQ1, we verified their interaction by the yeast two-hybrid system through truncating the cTP and a transmembrane domain of both HHL1 and SOQ1, and the growth of yeast cells on selective medium confirmed that HHL1 interacts with SOQ1 (Fig. 2*B*). Furthermore, we conducted bimolecular fluorescence complementation (BiFC) assays in Arabidopsis protoplasts. Co-expressing nYFP-tagged HHL1 and cYFP-tagged SOQ1 resulted in strong fluorescence in the chloroplasts of Arabidopsis protoplasts, while there was no fluorescence in Arabidopsis protoplasts cotransformed with nYFP-HHL1 and cYFP or nYFP and cYFP-SOQ1 (Fig. 2*A*). In addition, we confirmed the interaction between HHL1 and SOQ1 by co-immunoprecipitation (Co-IP) using transgenic Arabidopsis plants expressing SOQ1-FLAG and purified HHL1 (17). The results showed that HHL1 interacted with SOQ1 (Fig. 2*C*). Together, these results confirmed that HHL1 interacts with SOQ1.

### The enhanced NPQ in the hhl1 mutant is related to SOQ1 activity

**Figure 3:**
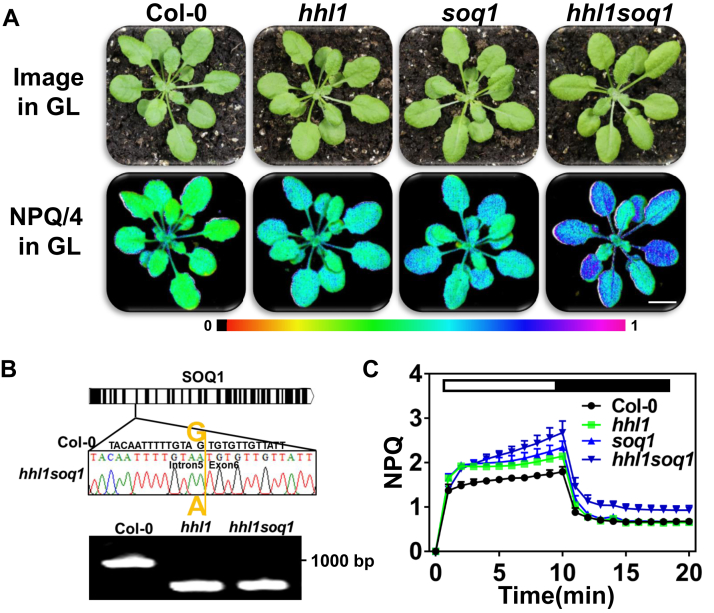
**Identification and phenotypic analysis of the *hhl1 soq1* double mutant, NPQ kinetic curves of 4-week-old Arabidopsis Col-0, *hhl1*, *soq1*, and *hhl1 soq1* plants under growth-light conditions, followed by induction with 500 μmol photons·m**^**−2**^**·s**^**−1**^**actinic light for 10 min and relaxation in the dark for 10 min; the saturation pulse was applied every 30 s.***A*, false-colored image of NPQ in plants following 10 min of actinic light induction. Scale bar, 1 cm. *B*, molecular identification of *hhl1 soq1*. *hhl1* is a transfer-DNA insertion mutant, which was identified by the three-primer method, and *soq1* contains a single point mutation in which the base before the sixth exon was mutated from G to A, as identified by sequencing. *C*, NPQ kinetics curves. Error bars represent SEM of six biological repeats. NPQ, nonphotochemical quenching.

To explore the genetic relationship between *HHL1* and *SOQ1*, we crossed the *hhl1* and *soq1* mutants to obtain the *hhl1 soq1* double mutant. The *soq1* mutant contains a single point mutation (14), and the *hhl1* mutant is a transfer-DNA insertion mutant (17) (Fig. 3, *A* and *B*). NPQ was higher in the *hhl1* and *soq1* single mutants than in the WT, whereas NPQ of the *hhl1 soq1* double mutant was higher than that of either single mutant (Fig. 3, *A* and *C*). The average NPQ values of Col-0, *hhl1*, *soq1*, and *hhl1 soq1* plants were approximately 1.78, 2.14, 2.35, and 2.67, respectively, when induced by actinic light for 10 min (Fig. 3*C*). After 10 min in the dark, the NPQ values of the WT and single mutants did not differ significantly (∼0.65–0.67), whereas the NPQ of *hhl1 soq1* was 0.93 (Fig. 3*C*). After high-light treatment, the NPQ values of *hhl1*, *soq1*, and *hhl1 soq1* were still higher than that of the WT after dark release (Fig. S2), suggesting that a light-dependent slow relaxation of NPQ occurred in these mutants.

Neither qE nor the trans-thylakoid pH gradient is associated with NPQ in the *hhl1* (Figs. 1 and S1) or *soq1* mutants (14). However, another important factor for NPQ induction in plants is the operation of the violaxanthin, antheraxanthin, and zeaxanthin cycle (20). Therefore, we analyzed pigment content in these lines. The total pigment contents per milligram of fresh weight of *hhl1* and *soq1* plants were not significantly different from those of the WT (Fig. S3, *A*–*D*). To determine whether the *hhl1* and *soq1* double mutation altered pigment composition, we performed high-performance liquid chromatography (HPLC) to analyze the pigment composition of the *hhl1 soq1* double mutant. Analyzing the carotenoid components among WT, *hhl1*, *soq1*, and *hhl1 soq1* plants (Fig. S3*E*) revealed that the NPQ in *hhl1 soq1* does not require zeaxanthin formation. This, combined with our finding that NPQ in the *hhl1* or *soq1* mutants is not related to qE, the trans-thylakoid pH gradient, or PsbS (Figs. 1 and S1) (14), suggests that the quenching observed in *hhl1* might be controlled by the same mechanism as in *soq1*.

As HHL1 is a PSII repair factor, it also participates in NPQ induction under high-light conditions. Conversely, SOQ1 is a NPQ suppressor; therefore, we reasoned that it might participate in PSII repair. To investigate this notion, we treated *hhl1* and *soq1* plants with high light and measured PSII activity (Fig. S4), the expression of photosynthesis and stress-related genes (Table S2), and the abundance of thylakoid membrane proteins in *soq1* (Fig. S5). We observed no obvious differences between *soq1* and the WT in these three measurements, which is different from the mutation of PSII photodamage repair factor HHL1 (17) (Figs. S4 and S5; Table S2), suggesting that SOQ1 does not function in PSII repair.

### HHL1 is required for the SOQ1-mediated inhibition of LCNP

**Figure 4:**
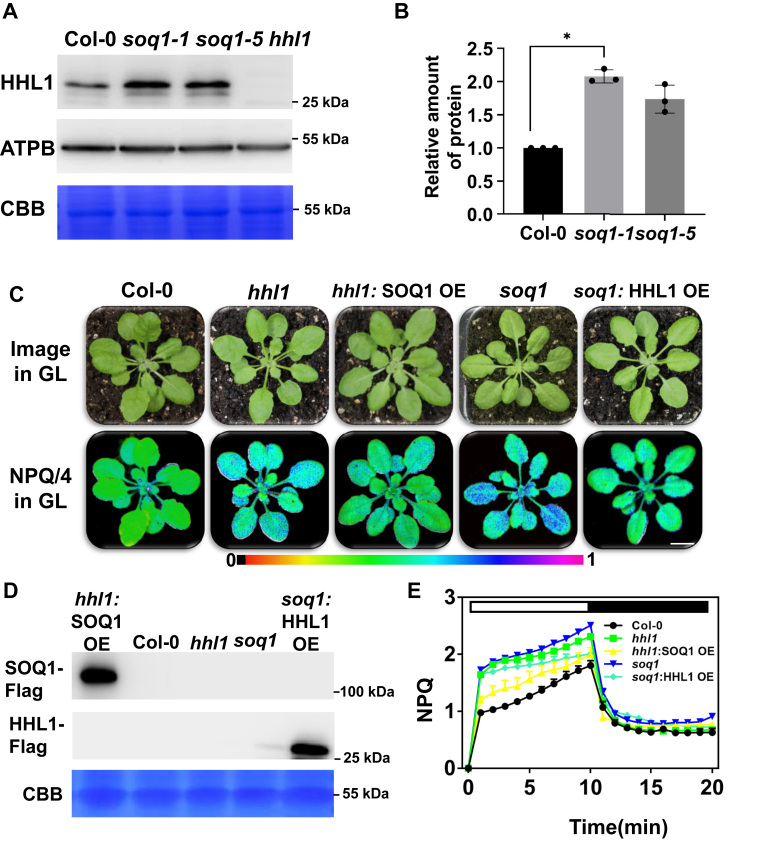
**Analysis of HHL1 protein levels in *soq1*.***A*, thylakoid membrane proteins were isolated from 14-day-old Arabidopsis Col-0, *soq1-1*, and *soq1-5* plants, separated by 15% SDS-PAGE, and subjected to immunoblotting with affinity-purified HHL antibody. ATPB (ATP synthase complex β subunit) and Coomassie Brilliant Blue (CBB) staining were used to ensure equal loading. *B*, quantitative analysis of HHL1 protein levels. Values are means ± SD of three replicates, (∗*p* < 0.05, Student’s *t* test). *C*, false-colored image of NPQ in plants after 10 min of actinic light induction. Scale bar, 1 cm. *D*, identification of proteins in *hhl1*:*SOQ1* OE and *soq1*:*HHL1* OE lines. *E*, NPQ kinetic curves of 4-week-old Arabidopsis Col-0, *hhl1*, *soq1*, *hhl1:SOQ1* OE, and *soq1*:*HHL1* OE under growth-light conditions, followed by induction with 500 μmol photons·m^−2^·s^−1^ of actinic light for 10 min and relaxation in the dark for 10 min; the saturation pulse was applied every 30 s. Error bars represent SEM of six biological repeats. HHL1, hypersensitive to high light 1; NPQ, nonphotochemical quenching; SOQ1, suppressor of quenching 1.

The abundance of HHL1 was higher in *soq1* than in the WT (Fig. 4, *A* and *B*), suggesting that HHL1 protein can compensate the loss of SOQ1. To test this possibility, we expressed *HHL1* under the control of the Cauliflower mosaic virus (CaMV) *35S* promoter in *soq1* and *SOQ1* under the control of the CaMV *35S* promoter in *hhl1*. The NPQ levels in both transgenic lines were intermediate between those of the WT and each of the single mutants (Fig. 4, *C* and *E*). Complementing *soq1* plants with *SOQ1* driven by the CaMV *35S* promoter restored NPQ to the WT level (14), whereas overexpressing *HHL1* significantly decreased NPQ levels in *hhl1* (Fig. 6, *A* and *C*). All these results suggest the functional synergy and divergence of HHL1 and SOQ.

**Figure 5:**
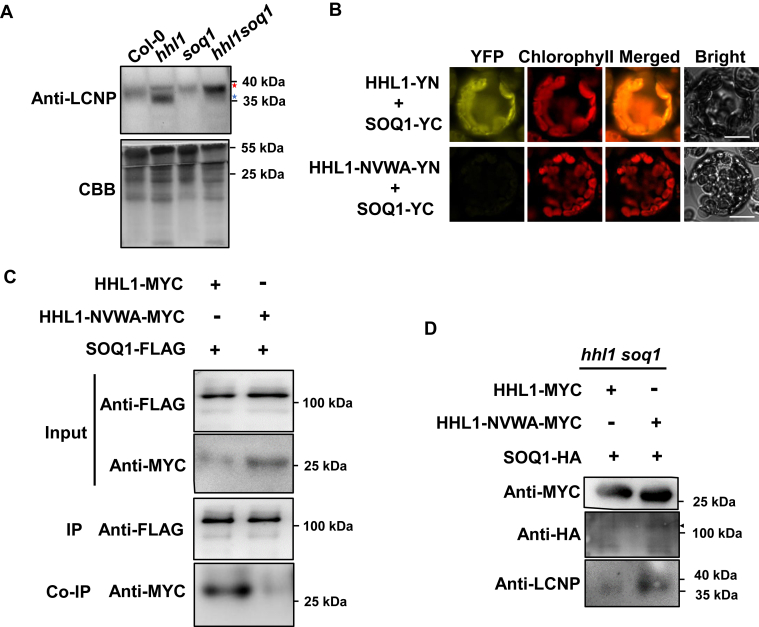
**Analysis of LCNP in the mutants, the function of the HHL1 VWA domain in the SOQ1 interaction, and the regulation of LCNP.***A*, After the high-light (500 μmol photons·m^−2^·s^−1^) and cold (4 °C) treatments in Col-0, *hhl1*, *soq1*, and *hhl1 soq1* for 5 h, protein was extracted and used for immunoblotting analysis. The anti-LCNP antibody was used to detect the protein level, and CBB was used for equal quantifying loading. Two independent biological replicates were performed with similar results. The *red star* represents the mobility protein band, the *blue star* represents the unmobility protein band. *B*, BiFC assay. The relevant vectors were cotransformed into Arabidopsis protoplasts, and fluorescence was observed by confocal microscopy. Scale bar, 10 μm. *C*, Co-IP assay. Protoplasts from 25-day-old transgenic Arabidopsis plants expressing *SOQ1-FLAG* were cotransformed with HHL1-nYFP (MYC tag) or HHL1-NVWA-nYFP. *D*, HHL1-NVWA-nYFP or HHL1-nYFP and SOQ1-nYFP were cotransformed into *hhl1 soq1* mesophyll protoplasts; anti-MYC, anti-HA, and anti-LCNP antibodies were used to detect the protein levels. The *arrow* represents the target band. At least two independent biological replicates were performed. BiFC, bimolecular fluorescence complementation; CBB, Coomassie Brilliant Blue; Co-IP, co-immunoprecipitation; HHL1, hypersensitive to high light 1; LCNP, plastidial lipoprotein; NPQ, nonphotochemical quenching; SOQ1, suppressor of quenching 1; VWA, von Willebrand factor type A.

**Figure 6:**
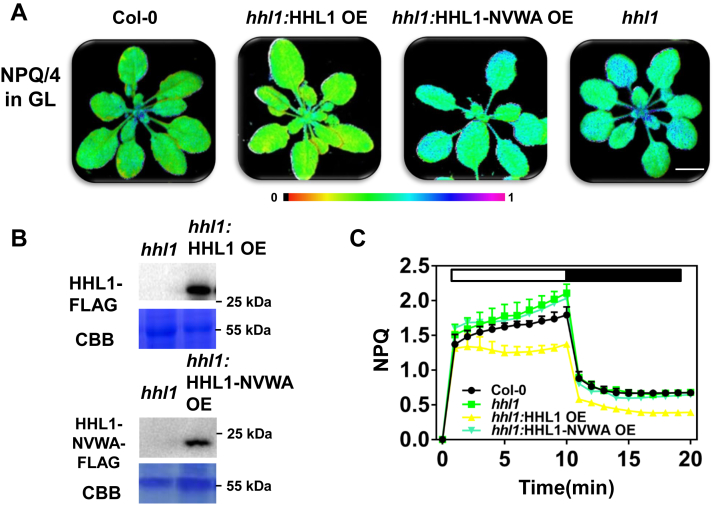
**Identification and phenotypic analysis of *hhl1*:*HHL1* OE and *hhl1*:*HHL1-NVWA* OE, NPQ kinetic curves of 4-week-old *hhl1*:*HHL1* OE and *hhl1:HHL1-NVWA* OE plants.** The plants were grown under growth-light conditions, induced with 500 μmol photons·m^−2^·s^−1^ actinic light for 10 min, and relaxed in the dark for 10 min; the saturation pulse was applied every 30 s. *A*, false-colored images of NPQ in plants after 10 min of actinic light induction. Scale bar, 1 cm. *B*, identification of proteins in *hhl1*:*HHL1* OE and *hhl1:HHL1-NVWA* OE lines. *C*, NPQ kinetics curve. Error bars represent SEM of six biological repeats. HHL1, hypersensitive to high light 1; NPQ, nonphotochemical quenching.

SOQ1 prevents qH by inhibiting LCNP, directly or through other proteins, and the apparent molecular mass of LCNP is slightly higher (1.5 kDa) in the *soq1* mutant background compared to the WT, as revealed by its altered mobility in a gel, which may be an oxidized form of LCNP (13, 15). According to our result about the nonredundant relationship of HHL1 and SOQ1, we assumed that HHL1 is involved in regulating LCNP; therefore, we examined LCNP accumulation in *hhl1* by immunoblot analysis. Cold and high-light conditions induce qH by altering of LCNP, which is regulated by SOQ1 (13). Therefore, we examined LCNP mobility under cold and high-light conditions in *hhl1*, *soq1*, and *hhl1 soq1* plants, the result showed that LCNP mobility was slightly altered in *hhl1* compared with the WT. Furthermore, the molecular mass of LCNP was higher in *soq1* and *hhl1 soq1* plants, and the quantity of LCNP was higher in *hhl1 soq1* than in *soq1* (Fig. 5*A*), suggesting that HHL1 is involved in regulating the modification of LCNP through SOQ1.

### The von Willebrand factor type A domain of HHL1 is required for its role in regulating LCNP and for NPQ

To understand the possible mechanism of HHL1 in regulating SOQ1-mediated modification of LCNP, we found that the C-terminal region of HHL1 includes one von Willebrand factor type A (VWA) domain (110–158 amino acids) based on SMART prediction (21), which mediates protein–protein interactions in integrins and extracellular matrix proteins (22). The roles of VWA domains in plants are unclear, but they are often involved in the formation of multiprotein complexes (23). To examine the function of the VWA domain of HHL1, we co-expressed the full-length HHL1 or a truncated version lacking the VWA domain with nYFP-tagged and cYFP-tagged SOQ1. The HHL1 protein without the VWA domain could not interact with SOQ1 (Fig. 5*B*). A Co-IP assay further confirmed the function of the HHL1 VWA domain in their interaction (Fig. 5*C*), indicating that the VWA domain of HHL1 mediates its interaction with SOQ1.

Then, we cotransformed HHL1 or HHL1 with the VWA domain deletion with SOQ1 into *hhl1 soq1* protoplasts. The LCNP level of *hhl1 soq1* protoplasts cotransformed with HHL1 and SOQ1 was similar to that in the WT (which contains LCNP protein with a lower molecular mass), but the LCNP level in *hhl1 soq1* protoplasts co-transformed with HHL1 lacking the VWA domain and SOQ1 was similar to that in the *hhl1 soq1* mutant (which contains LCNP protein with a higher molecular mass) (Fig. 5*D*), suggesting that the VWA domain of HHL1 participates in regulating the modification of LCNP through mediating the interaction of HHL1 with SOQ1. In addition, the β-propeller NHL domain of SOQ1 functions as substrate binding and protein–protein interactions and is essential for qH suppression (16). The C-terminal fragment of SOQ1 containing 159 residues, hereafter referred to as the CTD, which can rescue the qH suppression function of SOQ (16). Interestingly, the BiFC and Co-IP assays indicate the NHL and CTD domains of SOQ1 are required for the interaction between HHL1 and SOQ1(Fig. S6).

Finally, we overexpressed the cDNAs encoding the complete HHL1 protein and the HHL1 protein without the VWA domain in the *hhl1* mutant background. We detected related proteins in each overexpressing lines (Figs. 4*D* and 6*B*). The NPQ value in this line was below the WT level (Fig. 6, *A* and *C*). By contrast, overexpressing the HHL1 protein lacking the VWA domain did not affect NPQ in the *hhl1* mutant background (Fig. 6). Therefore, complementing *hhl1* plants with full-length HHL1 cDNA completely rescued the NPQ phenotype, whereas the HHL1 protein lacking the putative VWA domain was unable to complement the NPQ phenotype, suggesting that the VWA domain is involved in HHL1-mediated NPQ.

## Discussion

Changes in light intensity and quality affect plant growth. Plants must quickly adjust their photosynthetic state to cope with different light environments (3). NPQ is an important part of the photoprotective mechanism of plants. NPQ can be induced in a few seconds to a few min, allowing plants to rapidly respond to sudden fluctuations in light intensity (9, 20). NPQ can be roughly divided into several components, qE, qZ, qT, qH, and qI, which have different response times and involve different proteins (5, 27). qE, qZ, qT, and qI have been extensively investigated, which have different response times and involve different proteins (24). However, only three factors (SOQ1, LCNP, and ROQH) were found to be related to qH (13, 14, 19), and the process and mechanism underlying qH are unclear. Our previous study reported that HHL1 involved in the repair/turnover of PSII core proteins under high-light conditions (17, 28), but the role of HHL1 in NPQ is unknown. This study demonstrates that HHL1 interacts with SOQ1 to regulate NPQ.

The yeast two-hybrid screen of HHL1, BiFC, and Co-IP assays revealed that SOQ1 interacts with HHL1 (Fig. 2). Furthermore, the *soq1* and *hhl1* mutants showed higher NPQ than the WT (Fig. S2). Having ruled out the other components of NPQ (Figs. 1, S1 and S3), we infer that HHL1 play negative roles in regulating qH, which is similar to SOQ1. Moreover, HHL1 was more abundant in *soq1* than in the WT (Fig. 4, *A* and *B*), suggesting that the NPQ in *hhl1* is related to SOQ1 activity. Last, overexpressing *HHL1* in the *hhl1* background reduced NPQ to below WT levels (Fig. 6), while overexpressing *HHL1* in the *soq1* background did not totally reduce NPQ to WT levels (Fig. 4), which suggested that these proteins have synergy functions in regulating qH. Therefore, we conclude that HHL1 synergy regulates NPQ by interacting with SOQ1. Meanwhile, the NPQ of the *hhl1 soq1* double mutant was higher than that of the *hhl1* and *soq1* single mutants (Fig. 3, *A*–*C*), suggesting that these two proteins have divergence functions in regulating qH. Overexpressing HHL1 reduced NPQ in *hhl1* to below WT levels (Fig. 6), but overexpressing HHL1 did not reduce NPQ to even WT levels in *soq1* (Fig. 4), suggesting that HHL1 and SOQ1 have divergence functions in regulating NPQ. Above all, HHL1 and SOQ have synergy and divergence functions in regulating NPQ. More interestingly, the VWA domain of HHL1 and the NHL–CTD domain of SOQ1 are required for the interaction between HHL1 and SOQ1 (Figs. 5, *B* and *C* and S6).

The result showed that the molecular mass of LCNP was higher in the *soq1* and *hhl1 soq1* mutants, and LCNP accumulation was higher in *hhl1 soq1* than in *soq1* (Fig. 5*A*), suggesting that HHL1 is required for the SOQ1-mediated inhibition of LCNP. Notably, the cleavage of SOQ1 occurs in stress and nonstress conditions by a protease in the lumen to regulate its activity, which would affect SOQ1 cleavage into distinct soluble forms, such as CTD domains forms, and the degradation subproducts may thus have a functional role in the process of other proteins such as LCNP (16). We found that the VWA domain of HHL1 and the NHL and CTD domain of SOQ1 are required for the interaction between HHL1 and SOQ1 (Figs. 5, *B* and *C* and S6), and the VWA domain mediates the function of HHL1, which promotes the SOQ1-mediated inhibition of LCNP (Fig. 5*D*), suggesting that HHL1 participates with SOQ1 (may relate to NHL and CTD domain) to regulate the modification of LCNP in coregulate function in pH. SOQ1 is essential for the negative regulation of qH through participating in redox transduction from the Trx-like domain to target protein(s) (possibly LCNP or through other proteins) (16). But HHL1 did not contain conserved cysteines, which means that it does not participate in its own redox-regulated way. Interestingly, HHL1 shares a close evolutionary relationship with the Thimet Oligopeptidase family (25), implying a role of HHL1 in SOQ1 cleavage.

**Figure 7:**
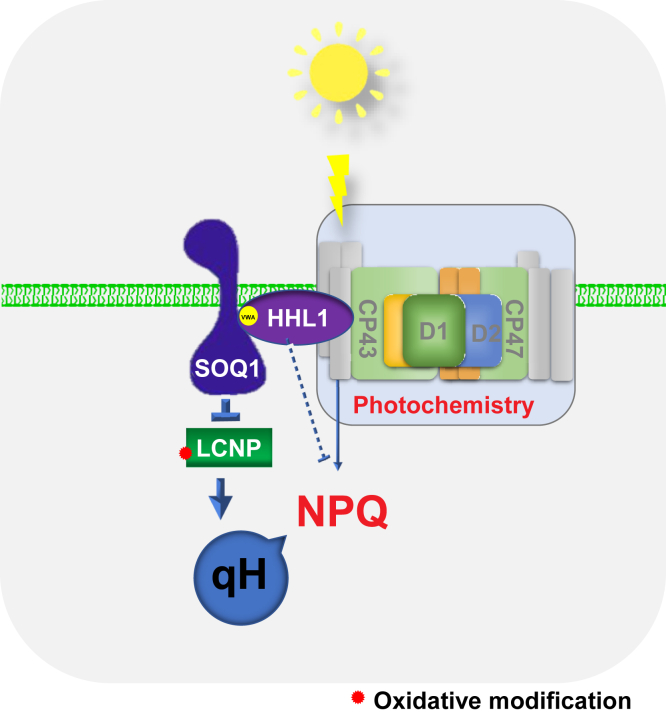
**Proposed working model of the synergy between HHL1 and SOQ1 in the regulation of NPQ.** On the lumenal side of the thylakoid membrane, HHL1 mainly interacts with SOQ1 through the VWA domain. Then, SOQ1 might provide reducing power to its target proteins, such as LCNP, and decrease oxidative modifications of LCNP, which is required to suppress qH (*left*). Meanwhile, HHL1 regulates NPQ *via* an unknown mechanism (*right*) to maintain normal plant growth. HHL1, hypersensitive to high light 1; LCNP, plastidial lipoprotein; NPQ, nonphotochemical quenching; SOQ1, suppressor of quenching 1; VWA, von Willebrand factor type A.

This study proposes a new mechanism of NPQ regulation that HHL1 interacts with SOQ1 by regulating the function of LCNP (Fig. 7). HHL1, as a unique factor, plays dual roles in the regulation of NPQ and PSII repair, thus building the relationship of NPQ and PSII repair in photoprotection.

## Experimental procedures

### Plant materials and growth conditions

The Arabidopsis (*A. thaliana*) *hhl1* (*SALK_129146C*), *soq1-1* (*N69919*), and *soq1-5* (*SAIL_7_D05*) mutants were obtained from the Eurasian Arabidopsis Stock Centre (uNASC). Plants were grown in soil in a growth chamber under controlled conditions (22 °C, 12-h-light/12-h-dark photoperiod, and 100 μmol photons·m^−2^·s^−1^ light intensity). Four-week-old plants were used for the experiments. For plants grown on agar plates, the seeds were sterilized in 75% ethanol (2 min) and 1.8% bleach (15 min) and sown on 1/2 Murashige & Skoog (1/2 MS) medium. Plants were grown in a growth chamber under controlled conditions (22 °C, 12-h-light/12-h-dark photoperiod, and 100 μmol photons·m^−2^·s^−1^ light intensity).

### Plasmid construction and plant transformation

To construct the *HHL1* or *SOQ1* overexpression lines, the *HHL1* and *SOQ1* coding sequences were amplified with gene-specific primers (Table S3). The truncated HHL1-NVWA protein was generated by deleting the sequence encoding amino acids 100 to 158 from HHL1 (Table S3). The open reading frame was cloned into pRi35S and inserted into pCAMBIA1301 to generate the *35S:HHL1-FLAG*, *35S:HHL1-NVWA-FLAG*, and *35S:SOQ1-FLAG* constructs. The different constructs were introduced into *Agrobacterium tumefaciens* strain EHA105 by heat shock. The transformants were then transformed into plants through the floral dipping method (26). Transgenic plants were selected on 1/2 MS agar plates with hygromycin.

### Yeast two-hybrid screening

HHL1 or SOQ1 without their cTP was cloned into the bait plasmid pGBK-T7 or pGAD-T7, respectively. The Matchmaker Gold Yeast Two-Hybrid system (Takara Bio) was used according to the manufacturer’s instructions.

### Bimolecular fluorescence complementation

Full-length bZIP63, HHL1, LQY1, and SOQ1 cDNAs were cloned into pUC-SPYNE or pUC-SPYCE, and the plasmids were cotransformed into Arabidopsis mesophyll protoplasts. The 514-nm and 488-nm laser lines with appropriate emission filters were used to image enhanced yellow fluorescent protein and chloroplast autofluorescence, respectively.

### Co-immunoprecipitation

The 10-day-old transgenic Arabidopsis seedlings expressing *SOQ1-FLAG* were used to extract total proteins with 1 ml IP buffer [10 mM Hepes (pH 7.5), 150 mM NaCl, 1 mM EDTA, 10% glycerol, 0.5% Triton X-100, and 1 pierce of protease inhibitor EDTA-free (Thermo)]. Then, 30 μl anti-FLAG M2 affinity gel (Cat# A2220; RRID: AB_10063035, Sigma) was added and incubated for 3 h at 4 °C. After washing three times with ice-cold IP buffer, the bound proteins were competitively eluted by 3× Flag Peptide (Beyotime). The presence of SOQ1-FLAG and HHL1 in the eluate (IP fraction) and input fraction was analyzed by SDS-PAGE and immunoblotting with anti-HHL1 (17) or HRP-conjugated anti-FLAG (Cat.#A8592; RRID: AB_439702, Sigma) antibodies.

The protoplasts from 25-day-old transgenic Arabidopsis plants expressing *SOQ1-FLAG* were cotransformed with HHL1-nYFP or HHL1-NVWA-nYFP, and the Co-IP assay was performed as described above. Anti-LCNP (Cat.#PAB211040, Orizymes), anti-MYC (Cat.#HT101, TransGen Biotech;), and anti-HA (Cat.#H3663, Sigma) antibodies were used.

### Chlorophyll fluorescence measurement

Chlorophyll fluorescence was measured at room temperature from attached, fully expanded rosette leaves or leaf discs of the same area using a Dual-PAM-100 (Walz) fluorimeter. Plants were dark-acclimated for 20 min, and NPQ was induced by treatment with 500 μmol photons·m^−2^·s^−1^ (red actinic light) for 10 min and relaxed in the dark for 10 min unless otherwise stated. Maximum fluorescence levels after dark acclimation (Fm) and throughout measurement (Fm’) were recorded after applying a saturating pulse of light.

### VIGS assay

The pTRV1 and pTRV2 plasmids are VIGS vectors based on the Tobacco rattle virus as described (29). To construct the pTRV2-PsbS vectors, *PsbS* cDNA was generated by RT-PCR with forward (ATGGCTCAAACCATGCTG) and reverse (ACCGATCATAGCAACACG) primers and inserted into the pTRV2 vector. The pTRV2-GFP vector was used as a negative control.

### Pigment analysis

Chlorophyll was extracted from 4-week-old plants with 80% acetone in 2.5 mM Hepes-KOH, pH 7.5. Carotenoids were extracted and analyzed as previously described using HPLC (13, 30). Pigments were identified by comparing retention times to references.

### Protein extraction and immunoblot analysis

Total proteins were extracted from leaves with extraction buffer (10 mM Hepes-KOH, pH 7.5, 150 mM NaCl, 5 mM EDTA, 0.5% [v/v] Triton X-100, 56 mM dithiothreitol, and protease inhibitor cocktail). Protein concentrations were determined using Folin-Phenol Reagent (Sangon Biotech) according to the manufacturer’s protocol. The proteins were separated by 15% SDS-PAGE and blotted onto polyvinylidene difluoride membranes (Millipore). Anti-HHL1 has been described previously (17), and anti-FLAG was purchased from Sigma. Antibodies against photosynthetic proteins were purchased from Agrisera (Cat.#:D1:AS05084;D2:AS06146;CP43: AS06110;CP47: AS05920;LHCA1: AS01005; ATPB: AS05085;PSBP: AS0614), and the signals were detected with SuperSignal West Pico Chemiluminescent Substrate (Thermo Scientific).

## Data availability

All the data contained within the manuscript.

## Supporting information

This article contains supporting information.

## Conflict of interest

The authors declare no conflict of interest with the contents of this article.
