# Supplementary material for: HHL1 and SOQ1 synergistically regulate nonphotochemical quenching in Arabidopsis
Source: J Biol Chem. 2023 Apr 5;299(5):104670. doi: 10.1016/j.jbc.2023.104670 (PMC10173003; doi:10.1016/j.jbc.2023.104670)
Supplement: Supporting Table S1–S3 [file mmc1.docx]

**Table S1. Proteins identified of HHL1 from yeast two-hybrid screening**

| **Gene** | **Molecular Function** |
| --- | --- |
| AT1G56500 | A thylakoid membrane protein with thioredoxin-like and beta-propeller domains located in the lumen and a haloacid-dehalogenase domain exposed to the chloroplast stroma. |
| AT4G00895 | ATPase, F1 complex, OSCP/delta subunit protein, proton-transporting ATP synthase activity, rotational mechanism |
| AT2G36250 | Encodes one of two FtsZ proteins, tubulin-like proteins, in Arabidopsis. It is involved in chloroplast division. Chloroplast fission, organelle fission. GTP binding, GTPase activity, protein binding. |
| AT3G01090 | ATP binding, kinase activity, kinase binding, phosphatase binding, protein binding, protein kinase activity, protein serine/threonine kinase activity. |
| AT1G73170 | P-loop containing nucleoside triphosphate hydrolases superfamily protein, ATP binding, hydrolase activity. |
| AT1G26720 | transmembrane protein. |
| AT5G04440 | RAP release 2, galactose-binding-like domain protein, putative (DUF1997). |
| AT1G56510 | TIR-NB-LRR protein that confers resistance to four races of Albugo candida.  The mRNA is cell-to-cell mobile. |

**Table S2 Gene expression analysis of Col-0, *soq1* and *hhl1***


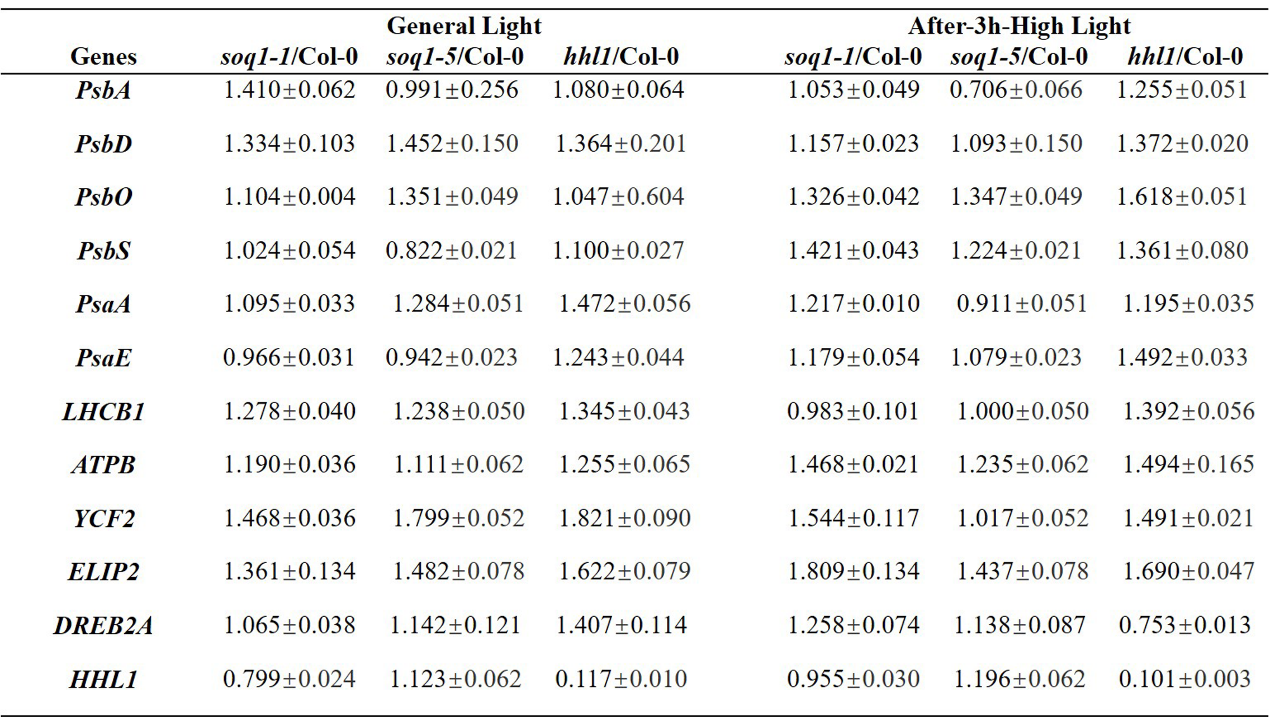


**Table S3 Primers Used in This Study.**

| **Primer** | **sequence (**5'→3'**)** |  |
| --- | --- | --- |
| Mutant identification | |  |
| *hhl1*-SALK_129146C-F | TAGCCATATCGAAACTCGGTG |  |
| *hhl1*-SALK_129146C-R | CGGTTCTGGTTCGGTTTAAAC |  |
| *soq1-1*-N69919-F | ATGCCATTGTTTCAGCAGATGCC |  |
| *soq1-1*-N69919-R | GGACCAGCATCCTTAAGAATT |  |
| *soq1-5*-SAIL_7_D05-F | TACAACTGTGCCAGAATTCCC |  |
| *soq1-5*-SAIL_7_D05-R | GCATTGGTGTTTTCCAAAATG |  |
| [**yeast two-hybrid**](javascript:;) **assay** | |  |
| HHL1^△CTP^-BD-F | CATGGAGGCCGAATTCATGGAGACGGCGGTACAGC |  |
| HHL1^△CTP^-BD-R  HHL1^△^-BD-F | GCCGCTGCAGGTCGACGGCCTTGGCTTTCTCATCCG  CATGGAGGCCGAATTCATGACTGCTAAGATCATGGTT |  |
| SOQ1^△^-BD-F | GGAGGCCAGTGAATTCATGGCATTGGTTGGAGCAAAAAGC |  |
| SOQ1^△^-BD-R | TCATCTGCAGCTCGAGGCGAGTACCTTGAAGCTG |  |
| **BiFC assay** | |  |
| HHL1-YN-F | GGGGATCCATGGAAGTGAGTATGTCT |  |
| HHL1-YN-R | AACTCGAGGGCCTTGGCTTTCTC |  |
| SOQ1-YC-F | GGCGCCGCCACTAGTGGATCCATGGCTTTGAAACTCACTTCTCCG |  |
| SOQ1-YC-R | CCCGGGAGCGGTACCCTCGAGGCGAGTACCTTGAAGCTGTAACC |  |
| HHL1-NVWA-R | TATCTTTGGTATCCTCTGAGTATATTTCGATATGGCTACAAACTT |  |
| HHL1-NVWA-F | AAGTTTGTAGCCATATCGAAATATACTCAGAGGATACCAAAGATA |  |
| SOQ1-NHL-CTD-YN | CCCGGGAGCGGTACCCTCGAGAGACGTGGCCAAACGTGGA |  |
| **VIGS** | |  |
| pTRV2-HHL1-F | GTGAGTAAGGTTACCGAATTCAAAGGTATGGCGGCGCGT |  |
| pTRV2-HHL1-R | GAGACGCGTGAGCTCGGTACCTTGTAGATATACTTCCCCAAGAGGTTAT |  |
| pTRV2-PsbS-F | GTGAGTAAGGTTACCGAATTCATGGCTCAAACCATGCTG |  |
| pTRV2-PsbS-R | GAGACGCGTGAGCTCGGTACC ACCGATCATAGCAACACG |  |
| **qPCR** | |  |
| PsbA-F | GCATAGCACTGAATAGGGAGCCG |  |
| PsbA-R | GCGACCTTGGATTGCTGTTGC |  |
| PsbD-F | GCGACCTTGGATTGCTGTTGC |  |
| PsbD-R | TGGTAGAACCTCCTCAGGGA |  |
| PsbO-F | CAGCCTCTCTCCAATCCAC |  |
| PsbO-R | GAGGTGGCAAGAGCGAATC |  |
| PsbS-F | CTCTTCAAACCCAAAACCAAAGCT |  |
| PsbS-R | CTCTTCAAACCCAAAACCAAAGCT |  |
| PsaA-F | CTACTTTGCCACCCACTGC |  |
| PsaA-R | TGAGTGCTTTAGGGCGTCC |  |
| PsaE-F | GGTTCAAGGCTAGTAGTCAGAG |  |
| PsaE-R | CTTGACCTTGGATCCTCTCTTT |  |
| LHCB1-F | CGTGACCATGCGTCGTACCGTC |  |
| LHCB1-R | CCT CAG GGAATGTGCATCCG |  |
| ATPB-F | CCT CAG GGAATGTGCATCCG |  |
| ATPB-R | GTTGAATCCACCACATAATCC |  |
| YCF2-F | TCTTTATTGGTTCTACCTCCTA |  |
| YCF2-R | TGCCTCCATTATGTTGTTG |  |
| ELIP2-F | CATCGCCATGGAGTTATCAAAG |  |
| ELIP2-R | TCATGAACCCTTTTGACTTTGC |  |
| DREB2A-F | CATGTTTGATGTCGATGAGCTT |  |
| DREB2A-R | ATTCCGTAGTTGAGGCTTTGTA |  |
| HHL1-F | AGCAGCGGCGGATGGTTTT |  |
| HHL1-R | GCATAGGAGGAGGTGTACGTTGATAC |  |
| ACTIN-F | GGTAACATTGTGCTCAGT GGTG |  |
| ACTIN-R | CTCGGCCTTGGAGATCCACATC |  |
| **Vectors construction** |  |  |
| HHL1-FLAG-F | ACAGAACCGACGACTACTAGTATGGAAGTGAGTATGTCTTTGAATGC |  |
| HHL1-FLAG-R GTCATCCTTGTAATCGTCGACGGCCTTGGCTTTCTCATCCG | |  |
| HHL1-NVWA-R | TATCTTTGGTATCCTCTGAGTATATTTCGATATGGCTACAAACTT |  |
| HHL1-NVWA-F | AAGTTTGTAGCCATATCGAAATATACTCAGAGGATACCAAAGATA |  |
| SOQ1-FLAG-F | ACAGAACCGACGACTACTAGTATGGCTTTGAAACTCACTTCTCCG |  |
| SOQ1-FLAG-R | GTCATCCTTGTAATCGTCGACGCGAGTACCTTGAAGCTGTAACC |  |
|  |  |  |
