# Supplementary material for: HHL1 and SOQ1 synergistically regulate nonphotochemical quenching in Arabidopsis
Source: J Biol Chem. 2023 Apr 5;299(5):104670. doi: 10.1016/j.jbc.2023.104670 (PMC10173003; doi:10.1016/j.jbc.2023.104670)
Supplement: Supporting Figures S1–S6 [file mmc2.docx]

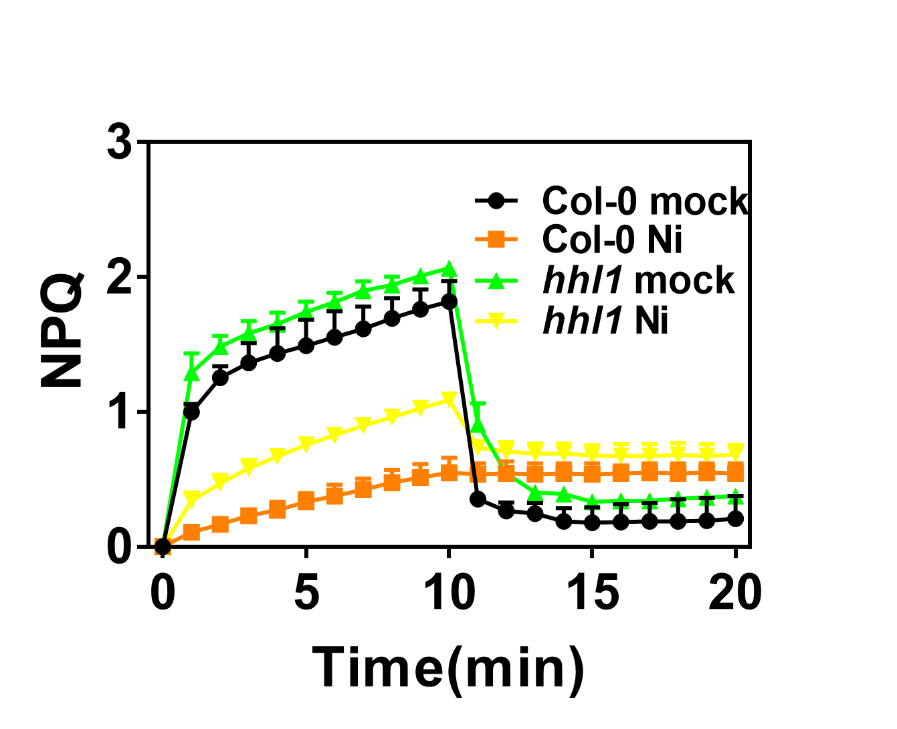


**Figure S1. NPQ kinetics in mock-treated *hhl1* plants and *hhl1* infiltrated with nigericin**

Leaves were collected from 4-week-old wild type (Col-0) and *hhl1* plants. Leaf discs were incubated in 2 mL bacteriocin reaction solution under a vacuum for 30–60 min. NPQ was detected after 10 min of photoinduction with 1,200 μmol photons·m^−2^·s^−1^ and 10 min of dark release. In the middle of the treatment, a saturation pulse was applied every 30 s to measure NPQ at the corresponding time. Error bars represent the SEM of six biological repeats.


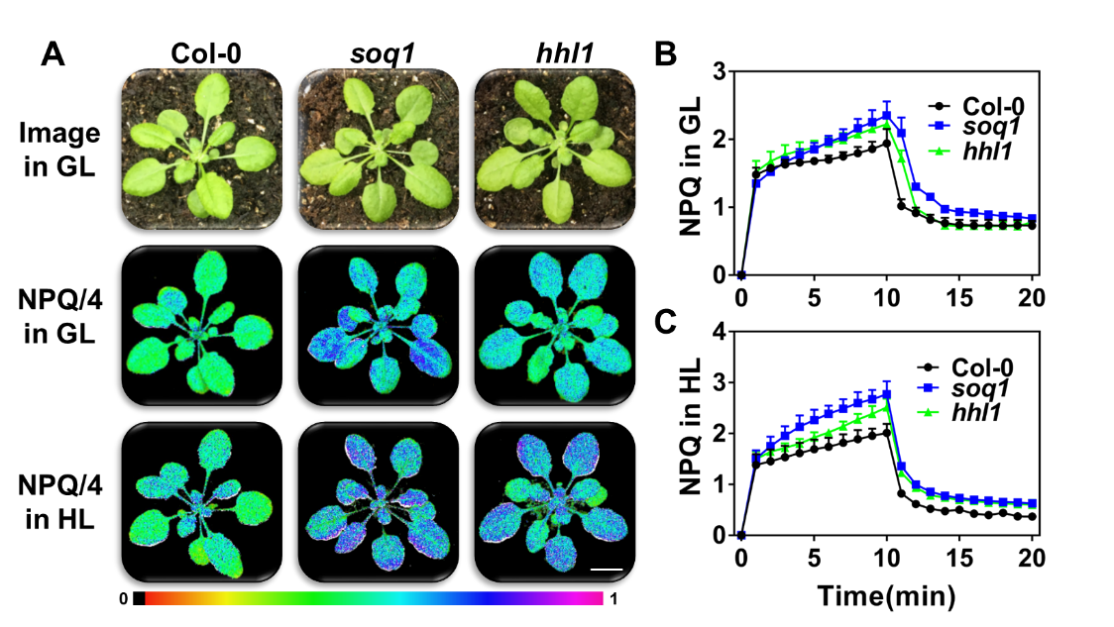


**Figure S2. NPQ induction in *soq1* and *hhl1* before and after high-light treatment**

Imaging analysis of NPQ parameters of 4-week-old Col-0, *hhl1*, and *soq1* plants before and after 3 h of high-light treatment (1,200 μmol photons·m^–2^·s^–1^). Imaging was performed using Imaging PAM. Before measurement, the plants were dark-adapted for 20 min. The plants were then treated with actinic light for 10 min and a dark release for 10 min. Detection was performed every 30 s. (A) The NPQ images were taken after 10 min of actinic light treatment. (B) NPQ dynamic curves of Col-0, *hhl1*, and *soq1* before high-light treatment. The actinic light (500 μmol photons·m^–2^·s^–1^) was turned on at time 0 and turned off after 10 min. (C) NPQ dynamic curves of Col-0, *hhl1*, and *soq1* after high-light treatment. The actinic light (1,300 μmol photons·m^–2^·s^–1^) was turned on at time 0 and turned off after 10 min. Scale bar, 1 cm. Error bars represent the SEM of six biological repeats.


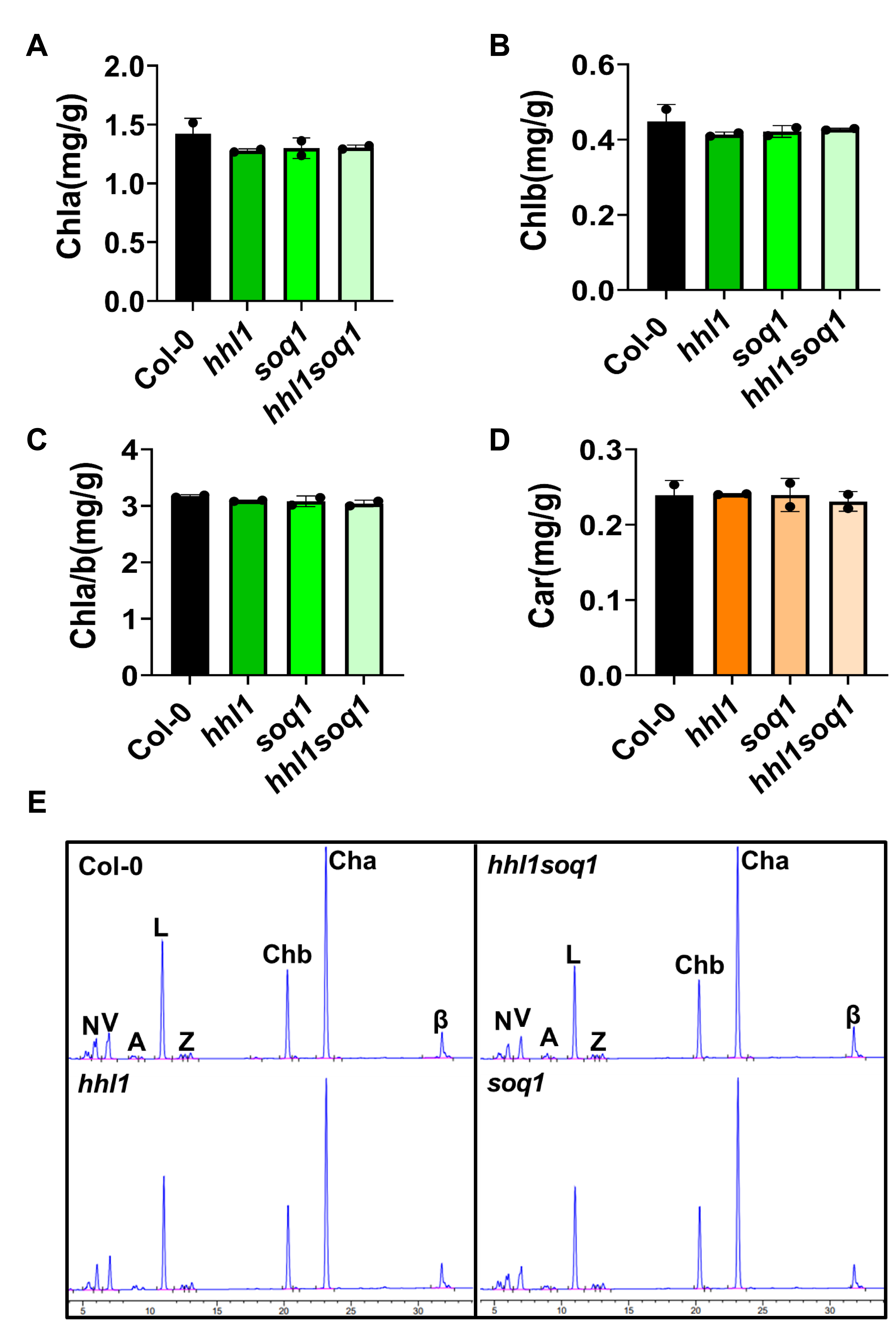


**Figure S3. Detection of total photosynthetic pigments in the *hhl1 soq1* mutant**

Detection of total photosynthetic pigments of Col-0, *hhl1*, *soq1*, and *hhl1 soq1*. (A) – (D) Leaves of equal weight from 4-week-old Arabidopsis plants under normal growth-light conditions were used to measure chlorophyll a (Chla), chlorophyll b (Chlb), the chlorophyll a/b ratio (Chla/b), and carotenoids (Car). Error bars represent SD of two biological repeats. (E) Leaves of equal weight from 4-week-old Arabidopsis plants under normal growth-light conditions were used to measure and compare the pigment profiles of the wild type (Col-0), *hhl1*, *soq1*, and *hhl1 soq1*. The retention time (min) and neoxanthin (N), violaxanthin (V), antheraxanthin (A), lutein (L), zeaxanthin (Z), β-carotene (β), chlorophyll a (Chla), and chlorophyll b (Chlb) are indicated.


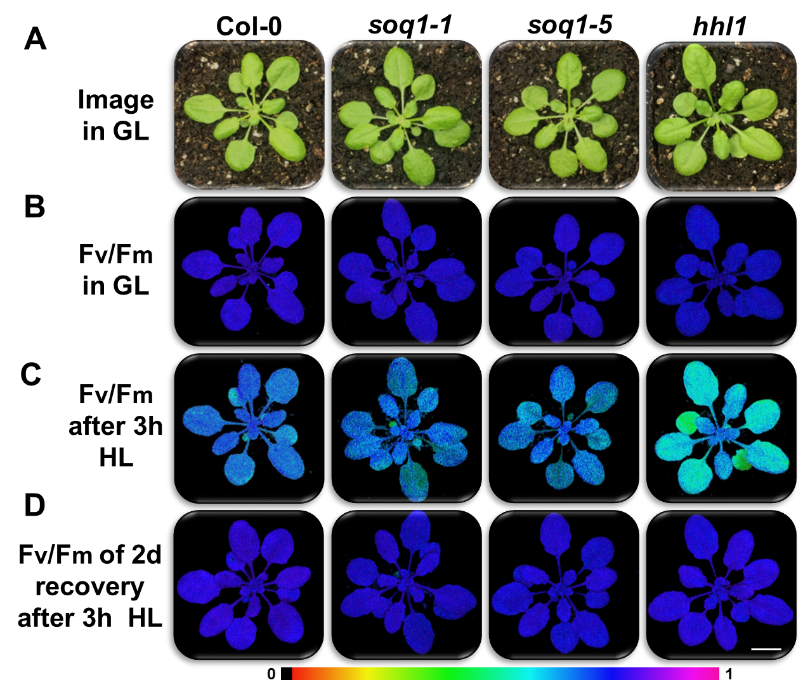


**Figure S4. F_v_/F_m_ imaging analysis of *soq1* before and after high-light treatment**(A) Images of 4-week-old wild-type Col-0, *soq1-1*, *soq1-5*, and *hhl1* plants under growth-light
conditions. (B) False-colored images representing F_v_/F_m_ of 4-week-old plants under growth-light conditions. (C) False-colored images representing F_v_/F_m_ of 4-week-old plants after a 3-h high-light treatment. (D) False-colored images representing F_v_/F_m_ of 4-week-old plants after a 3-h high-light treatment and a 2-d recovery period. Scale bar, 1 cm. GL, growth light (~100 μmol photons·m^–2^·s^–1^); HL, high light (~1,200 μmol photons·m^–2^·s^–1^). Error bars represent SEM of six biological repeats.


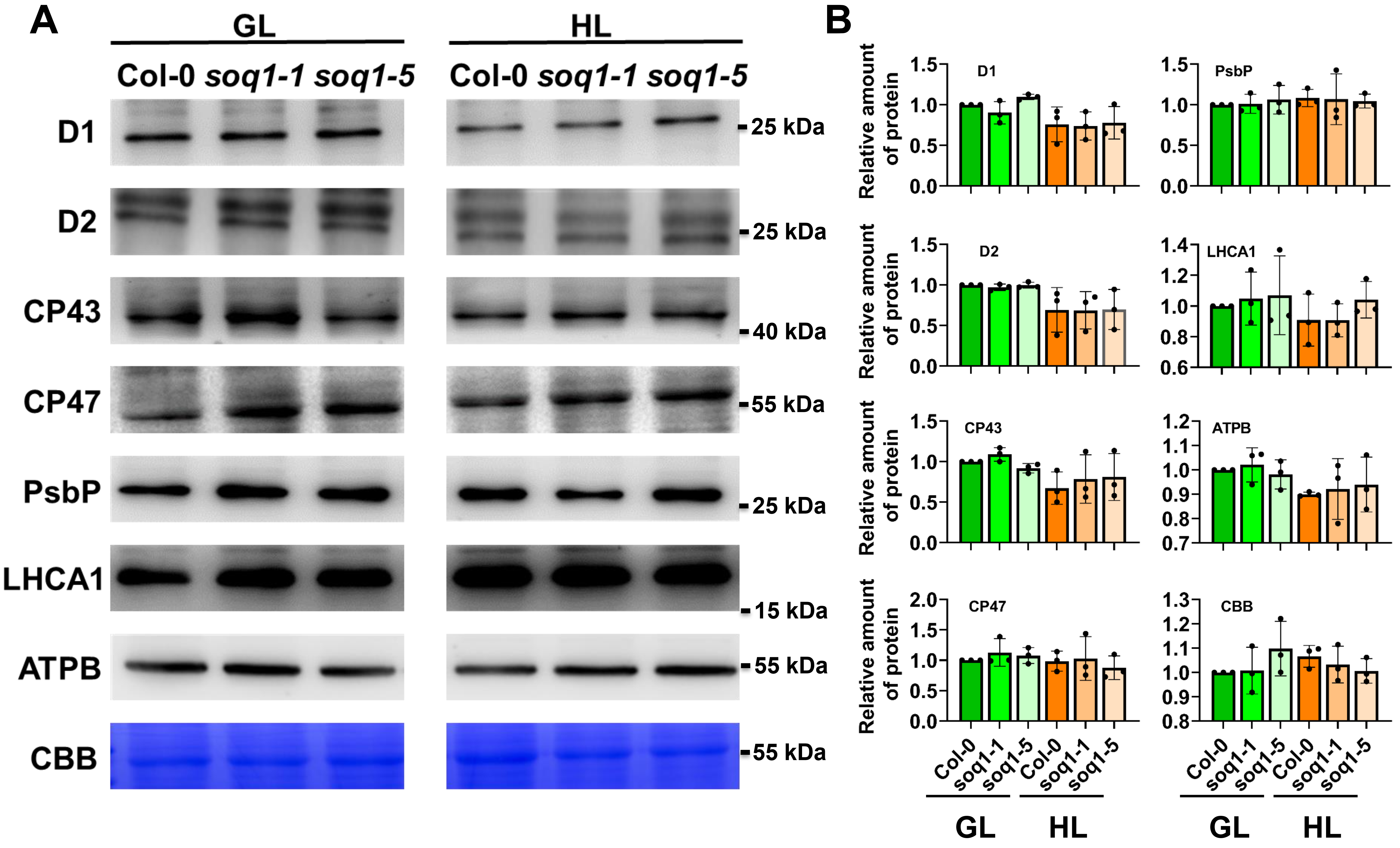


**Figure S5. Analysis of thylakoid protein accumulation in *soq1* before and after high-light treatment**

(A) Thylakoid membrane proteins from Col-0, *soq1-1*, and *soq1-5* plants were separated by 15% SDS-urea-PAGE, electroblotted onto PVDF membranes, and probed with antisera against known thylakoid membrane proteins obtained from Agrisera. GL, growth light; HL, after a 2-d high-light treatment. (B) The

quantitative analysis of the signal in A. Error bars represent SD of three biological repeats.


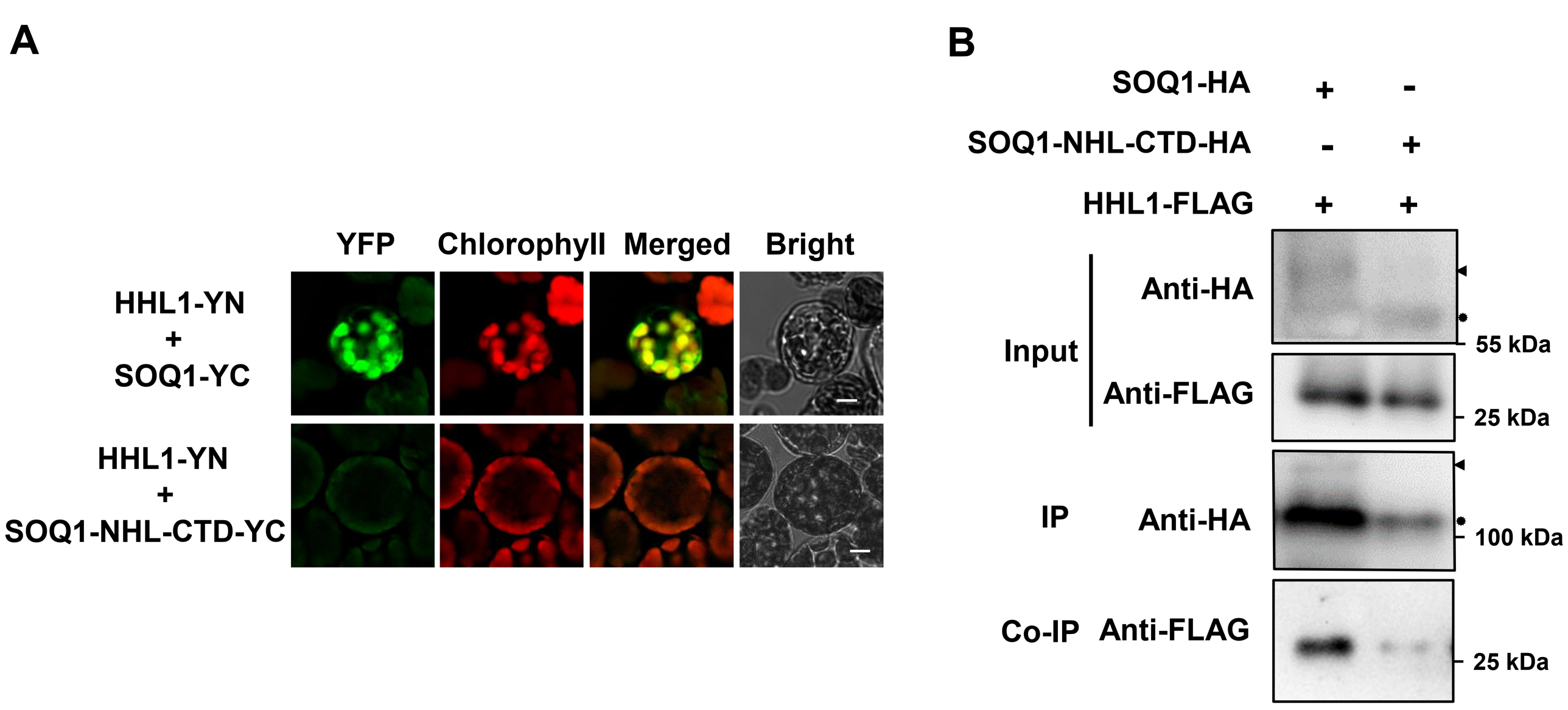


**Figure S6. The function of the NHL1 and CTD domains of SOQ1 in its interaction with HHL1.**

(A) BiFC assay. The relevant vectors were co-transformed into Arabidopsis protoplasts, and fluorescence was observed by confocal microscopy. Scale bar, 10 μm. (B) Co-IP assay. The protoplasts from 25-day-old transgenic Arabidopsis plants expressing *HHL1-FLAG* were co-transformed with SOQ1-HA or SOQ1-NHL-CTD-HA. Two independent biological replicates were performed with similar results. The arrow represents the SOQ1 band, and the dot represents the SOQ1-NHL-CTD band
